# Fatty acid profiles of the main lipid classes of green seaweeds from fish pond aquaculture

**DOI:** 10.1002/fsn3.511

**Published:** 2017-08-27

**Authors:** Carlos Cardoso, Andrea Ripol, Cláudia Afonso, Margarida Freire, João Varela, Hugo Quental‐Ferreira, Pedro Pousão‐Ferreira, Narcisa Bandarra

**Affiliations:** ^1^ Division of Aquaculture and Upgrading (DivAV) Portuguese Institute for the Sea and Atmosphere (IPMA, IP) Lisbon Portugal; ^2^ CIIMAR Interdisciplinary Centre of Marine and Environmental Research University of Porto Porto Portugal; ^3^ Centre of Marine Sciences University of Algarve Faro Portugal; ^4^ Aquaculture Research Station Olhão (EPPO) Portuguese Institute for the Sea and Atmosphere (IPMA, IP) Olhão Portugal

**Keywords:** fatty acid composition, fish pond aquaculture, green seaweed, lipid classes, nutritional composition

## Abstract

The lipid composition of five species of green seaweeds (*Chaetomorpha linum*,* Rhizoclonium riparium*,* Ulva intestinalis*,* Ulva lactuca*, and *Ulva prolifera*) grown in fish pond aquaculture systems was studied. In particular, the overall fatty acid (FA) profile and the FA profile of each main lipid class found in these seaweed species were thoroughly analyzed. It was found that every seaweed had a specific FA profile, whose specificities were rendered more obvious with the study of the FA profile per lipid class. However, between *U. lactuca* and *U. intestinalis*, there were only minor differences. Nonetheless, it was possible to identify significant differences between the palmitic acid content in the phospholipid (PL) and glycolipid (GL) classes of each seaweed. A clear distinction between the FA profiles of *R. riparium* and *C. linum*, which belong to the Cladophorales order, and those of *Ulva* genus, Ulvales order, was also determined. Moreover, there were also differences among lipid classes, yielding large contrasts between PLs + GLs and triacylglycerols (TAGs) as well as between monoacylglycerols (MAGs) and free fatty acids (FFAs). This study also found evidence supporting the location of particular FAs in specific TAG positions. FA profiles have the potential to be used as a chemotaxonomic tool in green seaweeds, providing a simple method to check authenticity of seaweed used as food.

## 1 INTRODUCTION

Seaweeds are still a largely undervalued marine resource. Besides, they can be produced in aquaculture systems, enabling a better control of their characteristics and composition. Indeed, they can be produced in separate ponds or as co‐products in fish and mollusk farming, for instance, in meagre (*Argyrosomus regius*) farming in earth ponds or in abalone (*Haliotis asinina*) farming in integrated multi‐trophic aquaculture (Largo, Diola, & Marababol, 2016). These aquaculture systems combine marine species that are commercially viable and environmentally sustainable on the basis of the concept that any waste consisting of uneaten feed, feces, and metabolic excretion of one species is an useful input for another species growth, thereby ensuring a natural self‐cleansing solution to pollution problems (Chopin et al., 2001). These polluting materials constitute a substantial problem in meagre farming, particularly there are components of fish feed with a low digestibility (Olim, 2012; Soler‐Vila & Moniz, 2012). Precisely, seaweeds may be able to operate as natural filters of nitrate and ammonia generated in meagre farming (Largo et al., 2016). This is environmentally valuable and may also provide some economic advantage. The composition and economic value of seaweeds may vary between species and, for a given species, parameters depend on abiotic/biotic conditions. Therefore, it is worthwhile to study the composition and properties of seaweeds from systems of fish pond aquaculture.

Though the lipid fraction has been less studied and typically does not surpass 5% of the dry seaweed matter in green seaweeds (El Maghraby & Fakhry, 2015; Kendel et al., 2015; Maehre, Malde, Eilertsen, & Elvevoll, 2014), it may comprise molecules with valuable bioactivities and may be a tool in differentiating seaweeds themselves and products derived from seaweeds, thereby enhancing traceability and reliability. Indeed, lipid profiling—such as overall and per lipid class fatty acid profiles—may be helpful in the assignment of algal taxonomic position and yield signature profiles for application in organic geochemistry and food studies (Rajasulochana, Krishnamoorthy, & Dhamotharan, 2010).

In particular, albeit variable, fatty acid profiles in seaweeds are usually rich in polyunsaturated fatty acids (PUFA), but with ω3 PUFA predominantly composed of shorter chain FAs, such as 16:4 ω3 and 18:4 ω3 (Kendel et al., 2015). There are also some species with significant amounts (on a dry matter basis) of eicosapentaenoic acid (20:5 ω3, EPA) (Dawczynski, Schubert, & Jahreis, 2007). Regarding health benefits, the ω3 PUFA class of FA is considered to play an important role in the prevention of cardiovascular and some autoimmune diseases, possessing anti‐tumoural and anti‐inflammatory properties (Dawczynski et al., 2007; Newton, 1996).

The aforementioned issues show that a study of the lipid fraction of a representative group of green seaweeds grown under fish pond aquaculture conditions is warranted. Precisely, this was the key objective of the performed analyses and data assessment carried out by this study: total FA profiles (for assessing FA quality and chemotaxonomic purposes); polar and apolar lipid distribution (chemotaxonomic purposes); and FA profiles of triacylglycerols (TAGs), monoacylglycerols (MAG), free fatty acids (FFAs), phospholipids (PLs), and glycolipids (GL) (FA quality and chemotaxonomic objectives).

## 2 EXPERIMENTAL

### 2.1 Cultivation conditions

At the Aquaculture Research Station, Olhão (EPPO), earth ponds with 0.2 ha and 2500 m^3^ in volume were used for meagre (*Argyrosomus regius*) experimental grow‐out from 10 g to 1 kg and, in some tanks, till 2.5 kg in fish weight. All ponds had constant water renovation, with a daily average of 30%, using pumped water from a reservoir connected directly to the Ria Formosa Lagoon. Dry feed is distributed to fish daily, starting with 2.3 (Winter, cold water, low feed consumption by the fish) and increasing progressively to 44 kg/day (Summer, warm water, high feed consumption by the fish), thereby reaching a total of 5,125 kg. No algicide (such as copper sulfate) was used during the grow‐out and the presence of macroalgae‐feeders like gilthead seabream, *Sparus aurata*, was low (less than 500 specimens per pond). Macroalgae biomass in the ponds was allowed to grow naturally until covering around 20% of water surface area and was collected weekly.

### 2.2 Samples

Samples of five species of green macroalgae (*Chaetomorpha linum*,* Rhizoclonium riparium*,* Ulva intestinalis*,* Ulva lactuca*, and *Ulva prolifera*) were collected manually and transported immediately in seawater to a nearby lab (<100 m). There was a single harvest in the second week of July. Approximately 200 g of each green seaweed (fronds) was collected by cutting and put in a seawater bucket. Each sample was thoroughly washed with seawater to eliminate any biofouling organisms. After washing, the frond samples were kept moist in a 20 L bucket and transported to the IPMA Lisbon Lab. Seaweeds were then finely minced in a GM200 Grindomix model mincer (Retsch GmbH, Haan, Germany) during 30 s and at a speed of 8,000 rpm. The processed biological material was frozen, freeze‐dried, and stored at −20°C.

### 2.3 Lipid extraction

Bligh & Dyer (1959) method was used for extraction of total lipid content from the fresh seaweeds. Briefly, 5 ml methanol:chloroform (2:1), 1 ml of saturated NaCl solution and 2 ml of chloroform were sequentially added and homogenized with 1 g of sample. After centrifugation (2,000*g* at 4°C for 10 min), organic phase was filtered through anhydrous sodium sulfate and evaporated in an RE 121 model rotary evaporator (Büchi, Flawil, Switzerland). Extractions were done in duplicate. Samples were stored at −20°C until further analyses.

### 2.4 Lipid class analysis

The main lipid classes were separated by analytical thin‐layer chromatography (TLC) in plates coated with 0.25 mm silica gel G and developed with a mixture of hexane:diethylether:acetic acid (50:50:2 by volume), based on the method described by Bandarra, Batista, Nunes, Empis, & Christie (1997). Extracted lipids were dissolved in chloroforom (10 mg/ml concentration). A mixture of standards (sigma chemical Co., St. Louis, Mo) was also prepared in chloroform with the same concentration. Specifically, glyceryltrioleate (TAG), glyceryl 1,3‐dipalmitate (diacylglycerol, DAG), DL‐α‐monoolein (MAG), oleic acid (FFA), L‐α‐phosphatidylcholine (PL), and monogalactosyl diacylglycerol (GL) were used. The samples and standards (10 μl) were applied to the plates and each plate was immersed in 102 ml of the elution mixture inside a developing chamber. The elution front was followed visually. After elution front reached the upper limit, plates were taken out from the chamber. The developed plates were then sprayed with 10% phosphomolybdic acid in ethanol (w/v). Identification of lipid classes (polar and apolar) was done by comparison with standards. Quantification was performed using a scanner and version 4.5.2 of Quantity One 1‐D Analysis software from Bio‐rad (Hercules, CA). There were always two replicates.

### 2.5 Lipid class separation for fatty acid analysis

The different lipid classes were fractionated using a preparative TLC. This involved applying 25 μl of a 50 mg/ml chloroform solution on several points of the TLC. The plate was placed in an elution vessel containing hexane:diethyl ether:acetic acid (50:50:2) and afterwards elution plates were sprayed with a 0.2% solution of 2′,7′‐dichlorofluorescein (Sigma, St. Louis, MO) in ethanol. Visualization was achieved in a cabinet II model UV chamber (CAMAG, Muttenz, Switzerland). Lipid fractions were identified using sigma standards (St. Louis, MO)—see section 2.4. There were always two replicates.

### 2.6 Fatty acid profile

Fatty acid methyl esters (FAMEs) were prepared by acid‐catalyzed transesterification using the methodology described by Bandarra et al. (1997). To 150 mg extracted crude fat present in a screw cap glass tube, 5 ml of a 5% acetyl chloride methanolic solution (prepared immediately before addition) were added. These glass tubes, after vigorous agitation, were placed in a hot bath (80°C) and left there 1 hour, in accordance with the method described by Lepage & Roy (1986), modified by Cohen, Vonshak, & Richmond (1988). After reaction completion, solution was cooled, diluted with 1 ml water and 2 ml n‐heptane and vigorously mixed, the last addition produced an organic phase that was filtered through anhydrous sodium sulfate. The resultant methyl esters were applied to a DB‐WAX (Agilent Technologies, Santa Clara, USA) capillary column (film thickness, 0.25 μm), 30 m × 0.25 mm i.d., integrated in a Varian Star 3800 CP gas chromatograph (Walnut Creek, CA), equipped with an auto sampler with a split injector (100:1) and a flame ionization detector, both at 250°C. The separation of the FAMEs was carried out with helium as the carrier gas and using a temperature program for the column starting at 180°C and increasing to 200°C at 4°C /min, holding for 10 min at 200°C, heating to 210°C at the same rate, and holding at this temperature for 14.5 min. FAMEs were identified by comparing their retention time with those of Sigma–Aldrich standards (PUFA‐3, Menhaden oil, and PUFA‐1, Marine source from Supelco Analytical). Analyses were always done in triplicate.

### 2.7 Statistical analysis

In order to test normality and variance homogeneity, the Kolmogorov‐Smirnov's test and Levene's *F*‐test, respectively, were used. Data fulfilled both of these parametric tests’ assumptions. The seaweed species (*C. linum*,* R. riparium*,* U. intestinalis*,* U. lactuca*, and *U. prolifera*) and the contrast between different lipid classes (TAG, PL+GL, MAG, and FFA) were the two studied factors. All statistically analyzed values were in percentage (% of total FA in the total FA comparison; % of total fat in lipid class distribution; and % of the FAs in each class in the respective lipid class). The parametric test, Tukey HSD, was done with STATISTICA 6, 2003 version (StatSoft, Inc., Tulsa, OK). For all statistical tests significance level (α) was 0.05. Whenever *p* was lower than α, statistical differences between species (total FA comparison; class distribution; specific class FA comparison) or between lipid classes for the same species (specific class FA comparison) were identified. In the former situation, lowercase letters were used, while, in the latter situation, uppercase letters were used.

## 3 RESULTS AND DISCUSSION

### 3.1 Seaweed fatty acid profile

The FA profile of the five studied seaweed species is presented in Table 1. These profiles encompass all fat present in all studied green seaweeds. A global comparison enables to point to two main aspects: *U. lactuca* and *U. intestinalis* FA profiles are very similar; all other profiles are quite different. Whereas *U. prolifera* is very rich in ω6 PUFA, *R. riparium* is much richer in ω3 PUFA. On the other hand, concerning ω3/ω6 ratio, the highest value is found for *C. linum*. In this species, total PUFA was lower than in *R. riparium* and *U. prolifera*. A high level of saturated FAs (SFA) contrasted with the low PUFA content in *C. linum*.

**Table 1:** Overall fatty acid profile (%) in the five studied green seaweed species

Fatty acid	*R. riparium*	*U. lactuca*	*U. prolifera*	*U. intestinalis*	*C. linum*
14:0	8.9 ± 0.1^b^	8.6 ± 0.0^b^	10.9 ± 0.0^c^	8.5 ± 0.0^b^	3.5 ± 0.3^a^
16:0	20.3 ± 0.2^b^	19.2 ± 0.1^a^	21.0 ± 0.2^b^	19.3 ± 0.0^a^	32.9 ± 0.4^c^
18:0	0.4 ± 0.0^ab^	0.3 ± 0.0^a^	0.2 ± 0.0^a^	0.3 ± 0.0^a^	0.6 ± 0.0^b^
Σ SFA	34.4 ± 0.1^a^	38.0 ± 0.0^b^	38.1 ± 0.2^b^	38.2 ± 0.2^b^	46.6 ± 1.2^c^
16:1 ω7 + ω9	6.4 ± 0.0^d^	1.2 ± 0.0^a^	1.6 ± 0.1^b^	1.2 ± 0.0^a^	4.3 ± 0.0^c^
18:1 ω7 + ω9	15.7 ± 0.2^c^	7.7 ± 0.0^a^	11.5 ± 0.2^b^	7.3 ± 0.0^a^	17.4 ± 0.2^d^
20:1 ω7 + ω9 + ω11	0.3 ± 0.0^a^	0.2 ± 0.0^a^	0.2 ± 0.0^a^	0.2 ± 0.0^a^	0.5 ± 0.0^a^
Σ MUFA	23.0 ± 0.2^c^	16.6 ± 0.1^b^	14.9 ± 0.1^a^	16.6 ± 0.1^b^	23.4 ± 0.2^c^
18:2 ω6	10.8 ± 0.4^c^	9.5 ± 0.2^bc^	22.0 ± 0.8^d^	8.1 ± 0.0^b^	2.1 ± 0.1^a^
20:4 ω6	0.9 ± 0.0^b^	1.8 ± 0.0^a^	1.7 ± 0.0^a^	1.8 ± 0.0^a^	0.1 ± 0.0^c^
16:3 ω3 + 16:4 ω3	4.0 ± 0.1^a^	10.6 ± 0.1^a^	8.7 ± 0.3^a^	11.0 ± 0.3^a^	0.9 ± 0.1^a^
18:3 ω3	10.5 ± 0.0^c^	0.1 ± 0.0^a^	0.2 ± 0.1^a^	0.1 ± 0.0^a^	4.1 ± 0.1^b^
18:4 ω3	0.4 ± 0.0^b^	0.2 ± 0.0^a^	0.5 ± 0.0^b^	0.2 ± 0.0^a^	2.3 ± 0.1^c^
20:4 ω3	0.1 ± 0.0^a^	0.1 ± 0.0^a^	0.5 ± 0.0^c^	0.1 ± 0.0^a^	0.3 ± 0.0^b^
20:5 ω3	2.7 ± 0.0^d^	1.6 ± 0.0^b^	2.2 ± 0.1^c^	1.7 ± 0.1^b^	0.6 ± 0.0^a^
22:5 ω3	2.1 ± 0.0^d^	1.4 ± 0.0^b^	1.9 ± 0.0^c^	1.5 ± 0.0^b^	0.7 ± 0.0^a^
22:6 ω3	0.4 ± 0.0^a^	0.2 ± 0.0^a^	0.2 ± 0.0^a^	0.2 ± 0.0^a^	0.4 ± 0.0^a^
Σ PUFA	33.1 ± 0.4^c^	26.8 ± 0.3^b^	39.0 ± 0.5^d^	26.0 ± 0.4^b^	12.2 ± 0.3^a^
Σ ω3	20.1 ± 0.1^c^	14.1 ± 0.0^b^	14.0 ± 0.3^b^	14.6 ± 0.4^b^	8.9 ± 0.2^a^
Σ ω6	12.0 ± 0.5^b^	12.1 ± 0.3^b^	24.7 ± 0.8^c^	10.7 ± 0.0^b^	2.4 ± 0.1^a^
Σ ω3/Σ ω6	1.7 ± 0.1^c^	1.2 ± 0.0^b^	0.6 ± 0.0^a^	1.4 ± 0.0^b^	3.7 ± 0.0^d^

Values are presented as average ± standard deviation. Different lowercase letters within a row correspond to statistical differences (*p* < .05).

A closer examination of data showed that EPA and docosahexaenoic acid (22:6 ω3, DHA) levels were always low in all seaweeds, not exceeding 3%–4% of the total FAs. The most abundant ω3 PUFA in *R. riparium* and *C. linum* was α‐linolenic acid (18:3 ω3). In the other three species, C16 ω3 FAs were the most abundant ω3 PUFA. The seaweed *U. prolifera* displays a high linoleic acid (18:2 ω6) content, 22.0 ± 0.8%, thus differing from other species of the same genus. The seaweed *C. linum* had a high concentration of 18:1, while this FA was less abundant in *U. lactuca* and *U. intestinalis*. Myristic (14:0) and palmitic (16:0) acids were the main SFA. Stearic acid (18:0) exhibited very low levels in the analyzed profiles. Though *C. linum* had high amounts of SFA, its myristic acid content was the lowest of all. On the other hand, its palmitic acid level was the highest among all studied species.

Regarding these results, there are similarities with other green seaweeds (Chopin et al., 2001), but there are also differences, including significant ones among studied species. Concerning similarities, palmitic acid has been claimed to be very abundant in seaweeds (Gressler et al., 2010). Other common traits of the FA profiles of green seaweeds are a high C18/C20 PUFA ratio and an abundance of C16 ω3 (Khotimchenko, Vaskovsky, & Titlyanova, 2002; Sato, 1975). These traits have been observed in the current study. On the other hand, there are differences between species due to specific aspects. This seems to make the study of the FA profiles a suitable scientific approach to distinguish between different green seaweed species. However, there are also important divergences in the FA composition of specimens of the same species collected from different locations, which jeopardizes the establishment of a straightforward link between a given FA profile and a particular green seaweed species. As an example, *U. lactuca* from North California coast in November presented 11% α‐linolenic acid, 22% stearidonic acid (18:4 ω3), 1% oleic acid (18:1 ω9), and 24% palmitic acid (Khotimchenko et al., 2002), while *U. lactuca* obtained from North Sea in September/October had 20% α‐linolenic acid, 8% stearidonic acid, 20% oleic acid, and 12% palmitic acid (van Ginneken, Helsper, de Visser, van Keulen, & Brandenburg, 2011). Accordingly, the application of lipidomics as a tool to differentiate green seaweed species may require a deeper analysis of the FA composition, involving analysis of the FA profile in each main lipid class (TAG, DAG, MAG, FFA, PL, and GL).

### 3.2 Seaweed lipid class distribution

In order to achieve the aforementioned objective, a first essential step is to determine the distribution of the fat substances into lipid classes. There was co‐elution of PL and GL. For this reason, it was chosen to group results into two major classes, polar and apolar (Table 2).

**Table 2:** Lipid class distribution (%) as determined by TLC of the five studied green seaweed species

Lipid class (%)	*R. riparium*	*U. lactuca*	*U. prolifera*	*U. intestinalis*	*C. linum*
Polar lipid	26.1 ± 3.9^a^	42.1 ± 4.0^b^	57.3 ± 8.5^c^	21.5 ± 1.2^a^	28.0 ± 0.8^a^
Apolar lipid	73.9 ± 3.9^c^	57.9 ± 4.0^b^	42.7 ± 8.5^a^	78.5 ± 1.2^c^	72.0 ± 0.8^c^

Values are presented as average ± standard deviation. Different letters within a row correspond to statistical differences (*p* < .05).

In *R. riparium*,* U. intestinalis*, and *C. linum*, the percentage of apolar lipids was higher than in the other seaweeds, thereby exceeding the 70% share of the total lipids. The highest percentage of polar lipids was measured in *U. prolifera*, 57.3% ± 8.5% of total lipids. With exception of this latter seaweed species, the values for the relative importance of polar and apolar lipids are within the ranges typically reported in the literature (Chopin et al., 2001).

### 3.3 Fatty acid profile of main lipid classes

The FA composition of the main lipid classes in the studied green seaweeds is shown in Tables 3 and 4. The PL (also including GL) and TAG profiles are found in the former table and the MAG and FFA are found in the latter. Because of the very low amount of DAGs in the lipid fraction of all seaweeds, it was not possible to determine the FA composition of this class. In the case of some seaweeds, there was poor separation of TAGs, MAGs, and FFAs from neighboring bands, thus leading to the exclusion of the FA profile determination for some classes and species.

**Table 3:** Phospholipid + glycolipid and triacylglycerol fatty acid profile (%) in the five studied green seaweed species

Fatty acid	Phospholipid + glycolipid classes					Triacylglycerol class				
	*R. riparium*	*U. lactuca*	*U. prolifera*	*U. intestinalis*	*C. linum*	*R. riparium*	*U. lactuca*	*U. prolifera*	*U. intestinalis*	*C. linum*
14:0	9.7 ± 0.1^bC^	14.1 ± 0.2^cB^	10.4 ± 0.6^bB^	10.5 ± 0.0^b^	2.4 ± 0.1^aC^	7.0 ± 0.1^cA^	n.d.	4.5 ± 0.4^bA^	n.d.	0.0 ± 0.0^aA^
16:0	30.8 ± 0.3^dC^	14.2 ± 0.3^aA^	23.6 ± 0.6^cB^	21.2 ± 0.0^b^	40.6 ± 1.0^eB^	17.2 ± 0.3^bB^	n.d.	7.3 ± 1.3^aA^	n.d.	25.6 ± 3.5^bA^
18:0	0.8 ± 0.0^bA^	0.4 ± 0.0^aA^	2.7 ± 0.0^eA^	1.6 ± 0.0^d^	1.3 ± 0.0^cA^	0.6 ± 0.0^aA^	n.d.	2.2 ± 0.7^aA^	n.d.	8.0 ± 1.6^bB^
Σ SFA	46.6 ± 0.4^abC^	44.1 ± 2.2^aAB^	42.2 ± 2.9^aB^	39.8 ± 0.0^a^	53.0 ± 1.2^bB^	27.2 ± 0.0^abB^	n.d.	19.8 ± 0.4^aA^	n.d.	37.4 ± 5.4^bA^
16:1 ω7 + ω9	4.8 ± 0.1^dA^	1.1 ± 0.0^bB^	0.8 ± 0.0^aA^	1.4 ± 0.0^c^	5.1 ± 0.2^eA^	6.4 ± 0.0^bC^	n.d.	2.9 ± 0.3^aB^	n.d.	4.1 ± 0.6^aA^
18:1 ω7 + ω9	12.3 ± 0.1^dC^	2.5 ± 0.1^aB^	11.7 ± 0.5^cAB^	7.6 ± 0.0^b^	12.8 ± 0.2^eA^	2.1 ± 0.0^aA^	n.d.	15.2 ± 0.4^bB^	n.d.	20.1 ± 0.1^cB^
20:1 ω7 + ω9 + ω11	0.6 ± 0.0^aB^	0.3 ± 0.0^aB^	0.7 ± 0.1^aA^	0.9 ± 0.0^b^	0.3 ± 0.1^aA^	0.0 ± 0.0^aA^	n.d.	0.6 ± 0.4^aA^	n.d.	1.7 ± 2.4^aA^
Σ MUFA	18.6 ± 0.1^dC^	4.3 ± 0.2^aB^	14.4 ± 2.0^cB^	10.4 ± 0.0^b^	19.5 ± 0.1^dA^	9.1 ± 0.0^aA^	n.d.	19.8 ± 0.4^bB^	n.d.	27.4 ± 3.6^bA^
18:2 ω6	7.2 ± 0.1^bB^	13.7 ± 0.2 ^dB^	19.4 ± 0.3^eB^	8.1 ± 0.0^c^	2.7 ± 0.1^aA^	8.3 ± 0.1^aC^	n.d.	35.3 ± 2.5^bC^	n.d.	3.2 ± 0.1^aB^
20:4 ω6	0.9 ± 0.0^bA^	0.1 ± 0.2^aA^	1.4 ± 0.0^cA^	1.7 ± 0.0^c^	0.4 ± 0.0^aC^	1.0 ± 0.0^bA^	n.d.	1.6 ± 0.3^bA^	n.d.	0.0 ± 0.0^aA^
16:3 ω3 + 16:4 ω3	3.4 ± 0.0^bA^	16.3 ± 0.3^eA^	8.8 ± 0.2^cB^	11.8 ± 0.0^d^	1.7 ± 0.0^aC^	3.3 ± 0.0^bA^	n.d.	8.3 ± 0.3^cB^	n.d.	0.0 ± 0.0^aA^
18:3 ω3	6.0 ± 0.0 ^dB^	0.3 ± 0.0^bB^	0.1 ± 0.0^aAB^	0.3 ± 0.0^b^	4.2 ± 0.1^cA^	13.4 ± 0.1^cC^	n.d.	0.3 ± 0.1^aB^	n.d.	6.4 ± 0.3^bC^
18:4 ω3	0.5 ± 0.0^bA^	0.3 ± 0.0^aB^	0.4 ± 0.0^aB^	1.6 ± 0.0^c^	1.7 ± 0.0^cA^	0.7 ± 0.0^aA^	n.d.	0.5 ± 0.1^aB^	n.d.	4.0 ± 0.5^bB^
20:4 ω3	0.1 ± 0.0^aB^	0.1 ± 0.1^aA^	0.4 ± 0.0^bB^	0.1 ± 0.0^a^	0.2 ± 0.0^abB^	0.2 ± 0.0^aC^	n.d.	0.6 ± 0.1^bB^	n.d.	0.0 ± 0.0^aA^
20:5 ω3	2.3 ± 0.1^aA^	1.6 ± 2.0^aA^	2.4 ± 0.2^aA^	2.6 ± 0.0^a^	1.5 ± 0.0^aB^	3.8 ± 0.1^bC^	n.d.	2.6 ± 0.5^bA^	n.d.	0.0 ± 0.0^aA^
22:5 ω3	2.4 ± 0.0^cB^	3.8 ± 0.1^dA^	2.1 ± 0.1^bC^	2.3 ± 0.0^bc^	0.7 ± 0.0^aC^	3.4 ± 0.1^cC^	n.d.	1.2 ± 0.2^bB^	n.d.	0.0 ± 0.0^aA^
22:6 ω3	0.7 ± 0.2^bA^	0.3 ± 0.1^aB^	0.2 ± 0.0^aB^	1.8 ± 0.0^d^	1.1 ± 0.1^cB^	0.3 ± 0.0^bA^	n.d.	0.2 ± 0.1^bB^	n.d.	0.0 ± 0.0^aA^
Σ PUFA	24.8 ± 0.4^bB^	39.9 ± 0.7^dA^	37.9 ± 0.9 ^dB^	32.0 ± 0.0^c^	16.0 ± 0.4^aA^	36.2 ± 0.3^bC^	n.d.	52.5 ± 4.0^cB^	n.d.	15.6 ± 2.8^aA^
Σ ω3	15.5 ± 0.4^bB^	23.9 ± 0.0^dA^	15.3 ± 1.2^bB^	20.6 ± 0.0^c^	11.1 ± 0.0^aB^	25.7 ± 0.1^bC^	n.d.	13.6 ± 1.5^aB^	n.d.	10.4 ± 0.1^aAB^
Σ ω6	8.6 ± 0.1^bB^	15.6 ± 0.6^dA^	22.6 ± 0.3^eB^	11.0 ± 0.0^c^	4.1 ± 0.3^aA^	9.9 ± 0.4^aB^	n.d.	37.7 ± 3.2^bC^	n.d.	5.2 ± 2.9^aA^
Σ ω3/Σ ω6	1.8 ± 0.0^bA^	1.5 ± 0.1^bA^	0.7 ± 0.1^aA^	1.9 ± 0.0^b^	2.7 ± 0.2^cA^	2.6 ± 0.1^aB^	n.d.	0.4 ± 0.0^aA^	n.d.	2.0 ± 0.4^aA^

n.d., not determined.

Values are presented as average ± standard deviation. Different lowercase letters within a row for each lipid class correspond to statistical differences (*p* < .05). Different uppercase letters between different lipid classes for each seaweed species (in both Tables 3 and 4) correspond to statistical differences (*p* < 0.05).

**Table 4:** Monoacylglycerol and free fatty acid profile (%) in the five studied green seaweed species

Fatty acid	Monoacylglycerol class					Free fatty acid class				
	*R. riparium*	*U. lactuca*	*U. prolifera*	*U. intestinalis*	*C. linum*	*R. riparium*	*U. lactuca*	*U. prolifera*	*U. intestinalis*	*C. linum*
14:0	9.6 ± 0.4^bC^	6.9 ± 1.6^abA^	5.3 ± 0.2^aA^	n.d.	n.d.	8.5 ± 0.0^cB^	6.8 ± 0.2^bA^	n.d.	n.d.	1.0 ± 0.0^aB^
16:0	31.4 ± 1.2^aC^	31.1 ± 6.6^aB^	41.4 ± 1.8^aC^	n.d.	n.d.	9.7 ± 0.0^bA^	6.6 ± 0.1^aA^	n.d.	n.d.	36.7 ± 0.0^cB^
18:0	2.1 ± 0.1^aB^	3.4 ± 0.8^aB^	7.4 ± 0.1^bB^	n.d.	n.d.	0.7 ± 0.0^aA^	0.6 ± 0.0^aA^	n.d.	n.d.	1.2 ± 0.0^bA^
Σ SFA	54.4 ± 1.5^aD^	50.8 ± 10.5^aB^	57.7 ± 2.2^aC^	n.d.	n.d.	23.0 ± 0.0^aA^	24.4 ± 0.4^aA^	n.d.	n.d.	44.0 ± 0.7^bAB^
16:1 ω7 + ω9	4.9 ± 0.1^bB^	0.0 ± 0.0^aA^	0.0 ± 0.0^aA^	n.d.	n.d.	6.9 ± 0.0^cD^	2.9 ± 0.0^aC^	n.d.	n.d.	6.5 ± 0.0^bB^
18:1 ω7 + ω9	5.1 ± 0.1^bB^	1.0 ± 0.2^aA^	2.9 ± 4.1^aA^	n.d.	n.d.	21.1 ± 0.1^bD^	17.0 ± 0.3^aC^	n.d.	n.d.	30.6 ± 0.1^cC^
20:1 ω7 + ω9 + ω11	0.0 ± 0.0^aA^	0.0 ± 0.0^aA^	0.0 ± 0.0^aA^	n.d.	n.d.	0.0 ± 0.0^aA^	0.4 ± 0.0^aC^	n.d.	n.d.	0.3 ± 0.2^aA^
Σ MUFA	10.0 ± 0.3^bB^	1.0 ± 0.2^aA^	6.0 ± 2.1^abA^	n.d.	n.d.	28.2 ± 0.0^bD^	20.6 ± 0.0^aC^	n.d.	n.d.	37.5 ± 0.0^cB^
18:2 ω6	2.2 ± 0.1^bA^	1.5 ± 0.2^aA^	2.0 ± 0.1^abA^	n.d.	n.d.	16.2 ± 0.1^cD^	14.9 ± 0.3^bC^	n.d.	n.d.	3.7 ± 0.0^aC^
20:4 ω6	1.1 ± 0.0^aB^	3.8 ± 0.8^bB^	2.8 ± 0.2^abB^	n.d.	n.d.	1.0 ± 0.0^bA^	2.8 ± 0.1^cB^	n.d.	n.d.	0.2 ± 0.0^aB^
16:3 ω3 + 16:4 ω3	3.8 ± 0.0^aB^	11.9 ± 2.8^bA^	2.8 ± 0.1^aA^	n.d.	n.d.	4.9 ± 0.0^bC^	10.7 ± 0.1^cA^	n.d.	n.d.	0.6 ± 0.0^aB^
18:3 ω3	1.8 ± 0.1^bA^	0.0 ± 0.0^aA^	0.0 ± 0.0^aA^	n.d.	n.d.	13.8 ± 0.2^cC^	0.3 ± 0.0^aB^	n.d.	n.d.	5.0 ± 0.0^bB^
18:4 ω3	0.8 ± 0.2^bA^	0.0 ± 0.0^aA^	0.0 ± 0.0^aA^	n.d.	n.d.	0.5 ± 0.0^aA^	0.4 ± 0.0^aC^	n.d.	n.d.	3.4 ± 0.3^bB^
20:4 ω3	0.0 ± 0.0^aA^	0.0 ± 0.0^aA^	0.0 ± 0.0^aA^	n.d.	n.d.	0.1 ± 0.0^aB^	0.2 ± 0.0^bA^	n.d.	n.d.	0.2 ± 0.0^bB^
20:5 ω3	3.2 ± 0.2^aB^	7.4 ± 5.8^aA^	2.8 ± 0.2^aA^	n.d.	n.d.	3.0 ± 0.0^bB^	3.3 ± 0.0^cA^	n.d.	n.d.	0.0 ± 0.0^aA^
22:5 ω3	0.8 ± 0.0^aA^	2.4 ± 2.3^aA^	0.0 ± 0.0^aA^	n.d.	n.d.	0.7 ± 0.0^aA^	1.8 ± 0.0^bA^	n.d.	n.d.	0.6 ± 0.0^aB^
22:6 ω3	0.5 ± 0.0^bA^	0.0 ± 0.0^aA^	0.0 ± 0.0^aA^	n.d.	n.d.	0.3 ± 0.0^aA^	0.4 ± 0.0^aB^	n.d.	n.d.	0.0 ± 0.0^aA^
Σ PUFA	15.6 ± 2.3^aA^	31.0 ± 9.9^aA^	13.9 ± 4.5^aA^	n.d.	n.d.	41.9 ± 0.5^cD^	36.8 ± 0.1^bA^	n.d.	n.d.	14.2 ± 0.2^aA^
Σ ω3	11.3 ± 1.1^abA^	21.7 ± 5.2^bA^	5.6 ± 0.3^aA^	n.d.	n.d.	23.6 ± 0.3^cC^	17.0 ± 0.1^bA^	n.d.	n.d.	9.9 ± 0.4^aA^
Σ ω6	3.7 ± 0.6^aA^	9.3 ± 4.7^aA^	8.4 ± 4.8^aA^	n.d.	n.d.	17.5 ± 0.2^bC^	19.0 ± 0.2^cA^	n.d.	n.d.	4.1 ± 0.2^aA^
Σ ω3/Σ ω6	3.1 ± 0.2^bB^	2.5 ± 0.7^abA^	0.8 ± 0.5^aA^	n.d.	n.d.	1.4 ± 0.0^aA^	0.9 ± 0.0^aA^	n.d.	n.d.	2.4 ± 0.2^bA^

n.d., not determined.

Values are presented as average ± standard deviation. Different lowercase letters within a row for each lipid class correspond to statistical differences (*p* < .05). Different uppercase letters between different lipid classes for each seaweed species (in both Tables 3 and 4) correspond to statistical differences (*p* < .05).

Within the PL + GL class, *C. linum* presented the highest SFA content (together with *R. riparium*) as well as the lowest PUFA content. Regarding MUFA, the lowest content was observed in *U. lactuca*, being the other seaweeds from the *Ulva* genus also poorer in MUFA than the seaweeds belonging to other genera. On the other hand, the highest percentage of ω3 PUFA in PLs and GLs was found in *U. lactuca*, displaying the other *Ulva* species also substantial amounts of ω3 PUFA. A similar situation was observed for ω6 PUFA except for the highest content being found in another *Ulva* species, *U. prolifera*. The seaweed *C. linum* exhibited the lowest percentages of both ω3 PUFA and ω6 PUFA. The relative richness in ω3 and ω6 PUFA in *U. prolifera* led to the lowest ω3/ω6 ratio, 0.7 ± 0.1. Contrastingly, *C. linum* displayed the highest ratio, 2.7 ± 0.2. However, most ω3 PUFAs had low abundance in this species. The main exceptions were the C18 ω3 PUFA. Highest DHA level was in *U. intestinalis*, but a low value (<2% of the total FAs), especially taking into account that it was determined in the PL + GL class. While there was no difference in the EPA content, all other ω3 PUFAs presented differences among seaweeds, namely, *U. lactuca* was the richest in C16 ω3 PUFA. For linoleic acid, highest value was determined in the PLs and GLs of *U. prolifera*, 19.4 ± 0.3%. On the other hand, the seaweeds *R. riparium* and *C. linum* were rich in C16 and C18 MUFA. Finally, whereas seaweeds from *Ulva* genus were rich in myristic acid (>10.0%), palmitic acid was much more abundant in *R. riparium* and, even more, in *C. linum*, reaching 40.6 ± 1.0%.

The FA profiles of the PLs and GLs had similarities with the global profiles of Table 1. There were also some differences. Namely, some FAs, such as palmitic acid, and the total SFA had different abundances in the PL + GL class. The PLs and GLs in *R. riparium* were poorer in ω3 PUFA than the total fat fraction in this seaweed. The opposite was observed in *U. lactuca* and *U. intestinalis*. In the case of *U. lactuca*, this can be mainly ascribed to the accumulation of C16 ω3 PUFA in the PL+GL class.

The differences between PLs and GLs and total fat may be ascribed to the other important lipid class in the studied green seaweed species, TAGs, which is the main group of apolar lipids. The comparison between the FA profiles of TAGs of different seaweeds conveys results similar to those found in PLs and GLs. For instance, as in PLs and GLs, *U. prolifera* had to the lowest ω3/ω6 ratio, 0.4 ± 0.0, and *C. linum* presented the highest ratio, 2.0 ± 0.4, or the highest content of linoleic acid was found in *U. prolifera*, 35.3% ± 2.5%. However, FA percentages in TAG differed significantly from those in PL + GL. For all seaweed species, TAGs were poorer in SFA than PLs and GLs. First and foremost, this was due to palmitic acid, but also myristic acid contributed for the SFA contrast between TAGs and PLs + GLs. Regarding MUFA, differences between TAGs and PLs + GLs were smaller. For ω3 PUFA and ω6 PUFA, differences were also less significant except for *R. riparium* in ω3 PUFA—higher in TAG class—and *U. prolifera* in ω6 PUFA—higher in TAGs. The latter was largely due to a high level of linoleic acid accumulation in the TAGs of *U. prolifera*. The former deviation resulted from a high level of α‐linolenic acid in *R. riparium* TAGs, 13.4% ± 0.1%.

The FA compositions of the MAG and FFA classes (Table 4) are related to each other since MAGs are formed from TAG (and DAG) by hydrolysis, which also generates FFAs. Accordingly, a joint analysis of the FAs in each of these two classes can provide valuable insight. It was observed that SFA and palmitic acid percentages were higher and MUFA and linoleic acid percentages were lower in the MAG than in FFA, thereby pointing to a preferential hydrolysis of MUFA and linoleic acid. Moreover, a global comparison involving all studied lipid classes shows that SFA were much more abundant in MAG and PL + GL than in TAG and FFA. Concerning other FAs, differences were circumscribed to particular species. For instance, PUFA, including both ω3 and ω6, and, particularly, α‐linolenic acid were higher in FFA class only in the case of *R. riparium*.

According to literature (Kendel et al., 2015), higher palmitic and SFA contents in PLs and GLs than in the total fat fraction were also observed for another green seaweed, *U. armoricana*. A lower level of ω3 PUFA in PLs (13.8% ± 0.1%) and even lower in GLs (8.5%) than in total lipids (23.9% ± 0.1%) was also found in this species by the same authors. This contrasts with other organisms, where ω3 PUFA, particularly very long chain ω3 PUFA (EPA, DHA), are typically more concentrated in the PL fraction and other polar lipids fractions (Mendoza Guzmán, de la Jara, Carmona‐Duarte, & Freijanes‐Presmanes, 2011), since EPA and DHA are structurally important FAs giving fluidity to cell membranes (Valentine & Valentine, 2004). Hence, *R. riparium* represents an uncommon situation characterized by higher ω3 PUFA content in TAGs than in PLs and GLs. In *U. lactuca*,* U. prolifera*, and *U. intestinalis*, there was no specific accumulation of α‐linolenic acid in PLs, thus differing from other algae (Kumari, Kumar, Reddy, & Jha, 2013). This may explain that though α‐linolenic acid is considered characteristic of the order Ulvales, reaching 10%–20% of the total FAs (Khotimchenko et al., 2002), its content in the studied Ulvales (genus *Ulva*) was low—PLs did not contribute much to the global α‐linolenic acid content.

The preferential hydrolysis of MUFA and linoleic acid over SFA and palmitic acid can be related to the selectivity of any lipase that remains active after harvest and during transport and storage of the seaweeds. This selectivity may lead to the formation of some FFAs and the relative concentration of certain FAs, such as palmitic acid, in the MAGs. Moreover, lipases may operate in a selective way owing to either chemical affinity or sensitivity to the position of the FA chain in the TAG. On the one hand, whereas ω3 PUFA such as DHA are very frequently bound at the 2‐position (*sn*−2) of TAG molecules, two other mid‐ or short‐chain FAs are in the lateral (1‐ and 3‐) positions (*sn*−1/3) (Schuchardt & Hahn, 2013). This makes the rupture of the ester bond of a long‐chain fatty acid by a lipase harder to achieve (Schuchardt & Hahn, 2013). On the other hand, the chemical structure of each FA, in particular, the number of double bonds, may be more important than position. Regarding the positional versus chemical structure selectivity hypotheses, the enrichment in palmitic acid in the MAG supports the regioselectivity hypothesis. This is a saturated FA and it is not very long, thus any structural selectivity against DHA hydrolysis would not apply to this FA. Moreover, a positional selectivity of the lipase implies that the palmitic acid (and other SFA) is more frequently bound at the 2‐position (*sn*−2) of TAGs in seaweeds. Precisely, it has been reported for other eukaryotic organisms a higher proportion of palmitic and other SFA in position *sn*−2 (Brockerhoff, Hoyle, Hwang, & Litchfield, 1968). It is also very interesting to note that according to this study that different MUFAs are most often found at *sn*−1/3. This agrees with the results of the current study. Therefore, the lipase responsible for the observed hydrolysis seems to display a predominantly regioselective action and the positioning of FAs in the green seaweed lipids does not differ much from that of other organisms.

Results seem to enable two main dividing lines: between Ulvales (*U. lactuca*,* U. prolifera*, and *U. intestinalis*) and Cladophorales (*R. riparium* and *C. linum*) and between *U. prolifera* and the group formed by *U. lactuca* and *U. intestinalis*. In particular, for the first dividing line, important discriminating parameters for the Ulvales are: low MUFA content in total fat, PL + GL, and MAG; low C18:1 content in total fat, PL + GL, and MAG; high C16 ω3 PUFA content in total fat and PL + GL; and low ω3/ω6 ratio in total fat. For the second dividing line, *U. prolifera* differs from the other species of the *Ulva* genus in: high C18:1 content in total fat and PL + GL; low MUFA content in total fat; high linoleic acid content in total fat and PL + GL; high PUFA content in total fat; high ω6 PUFA in total fat and PL + GL; and low ω3/ω6 ratio in total fat and PL + GL. Regarding this second contrast, it is worth noting that, according to phylogenetic studies on the basis of genetic analysis, *U. lactuca* and *U. intestinalis* (also known as *Enteromorpha intestinalis*) are nearer to each other than to *U. prolifera* (also known as *Enteromorpha prolifera*) (Hayden et al., 2003). Therefore, FA profiles seem to be usable as a chemotaxonomic tool in green seaweeds. Given the simplicity of the FA determination methodology, this can provide a quick and practical route for the verification of seaweed identity in slightly processed foods—for instance, all *Ulva* species are edible (Edwards et al., 2012), being seaweed dried and finely minced. Nonetheless, more research covering multiple influential aspects, such as season, geographical location, cultivation methods, and others, must be carried out in order to consolidate this possibility.

Finally, it should be noted that seaweed quality as a source of essential FAs could be monitored through the calculation of critical ratios in the PL + GL fraction as well as in total fat, such as, ω3/ω6 ratio, ω3(C20 + C22)/ω3(C16 + C18) ratio, and the atherogenicity (AI) and thrombogenicity (TI) indices:(Senso, Suárez, Ruiz‐Cara, & García‐Gallego, 2007)

$$\begin{array}{cc} \text{AI}= & [(4\times C14:0)+C16:0+C18:0]/(\sum \text{MUFA}+\sum \omega 6\text{PUFA}\\ & +\sum \omega 3\text{PUFA}) \end{array}$$

$$\begin{array}{cc} \text{TI}= & (C14:0+C16:0+C18:0)/(0.5\times \sum \text{MUFA}+0.5\times \sum \omega 6\text{PUFA}\\ & +3\times \sum \omega 3\text{PUFA}+\omega 3/\omega 6\text{ratio)} \end{array}$$

Regarding these FA quality parameters, different seaweeds presented the best levels: highest ω3/ω6 ratio in *C. linum*'s total fat (and PL + GL); highest ω3(C20 + C22)/ω3(C16 + C18) ratio in *U. prolifera*'s total fat; and lowest AI and TI in *R. riparium*'s total fat.

## 4 CONCLUSIONS

The fish pond aquaculture production system showed to enable the rearing of meagre and the growth of different green seaweed species with specific fat fraction characteristics. Indeed, there was a clear distinction between the FA profiles (total FA and per lipid class) of *R. riparium* and *C. linum*, which belong to the Cladophorales order, and those of *Ulva* genus, Ulvales order. Moreover, every seaweed had a specific FA profile, whose specificities were rendered more obvious with the study of the FA profile per lipid class. However, between *U. lactuca* and *U. intestinalis*, there were only minor differences. On the other hand, *U. prolifera* differed from the other species of the *Ulva* genus. Furthermore, it was possible to identify significant differences between the palmitic acid content in the PL + GL class of each seaweed. Hence, FA profiling may offer a simple and practical tool for distinguishing among seaweed species, for instance, detecting non‐edible species in dried and minced seaweed‐based foods. Important differences were found among lipid classes, yielding large contrasts between PLs + GLs and TAGs as well as between MAGs and FFAs. This study also found evidence supporting the location of particular FAs in specific TAG positions. There are still many unknown aspects, such as the effects of season, wild versus cultured seaweeds, geographical location and other factors on the FA profiles, thus warranting further study.

## CONFLICT OF INTEREST

No conflict of interest involving any of the authors.
